# Optimizing induction chemotherapy regimens for radiotherapy in patients with locoregionally advanced nasopharyngeal carcinoma

**DOI:** 10.1002/cam4.5707

**Published:** 2023-03-05

**Authors:** Ying Li, Jianping Bi, Guoliang Pi, Hanping He, Yanping Li, Dandan Zheng, Zecheng Wei, Guang Han

**Affiliations:** ^1^ Department of Radiation Oncology, Hubei Cancer Hospital, Tongji Medical College Huazhong University of Science and Technology Wuhan Hubei China; ^2^ Department of Radiation Oncology University of Rochester Rochester NY USA; ^3^ Neurosurgery department, The fifth hospital of Wuhan Wuhan Hubei China

**Keywords:** induction chemotherapy, nasopharyngeal carcinoma, optimal cycles, tumor deformation, volumetric change

## Abstract

**Background and Purpose:**

The optimal number of cycles of induction chemotherapy (IC) in locoregionally advanced nasopharyngeal carcinoma (LANPC) remains unresolved. This study aimed to quantitatively assess the changes in gross tumor volumes (GTVs) and to select the most optimal number of IC cycles.

**Methods:**

We analyzed 54 patients who received a three‐cycle IC before commencing radiotherapy, with the tumor and nodal responses assessed by a CT scan before IC and after each IC cycle. The gross tumor volumes of the nasopharynx primary lesion (GTV_T), involved retropharyngeal lymph node (GTV_RP), and involved cervical lymph node (GTV_N) were contoured on each scan. The volume change following each IC cycle was evaluated with Wilcoxon signed‐rank test. The three‐dimensional vector displacements of target centers were also calculated and compared.

**Results:**

The volume reductions of GTVs following IC varied across different patients and showed different trends for the three GTV types. GTV_T and GTV_RP did not display further volume reduction after two IC cycles, whereas GTV_N showed monotonic volume decreases. For GTV_T and GTV_RP following the three IC cycles, the total volume reduction relative to the initial volume before IC was 12.0%, 22.5%, and 20.1% and 26.0%, 44.1%, and 42.2%, respectively. In contrast, for GTV_N, continuing volume reduction was observed with a total reduction of 25.3%, 43.2%, and 54.7% following the three cycles, and the reductions were all significant. Average displacements of the GTVs were <1.5 mm in all directions; their average three‐dimensional displacements were 2.6, 4.0, and 1.7 mm, respectively. Acceptable toxicity was observed in most patients.

**Conclusion:**

This study supports two cycles of IC before radiotherapy for patients with LANPC if the initial metastatic cervical lymph node volume is not dominating. Otherwise, three cycles of IC is recommended to further reduce the cervical node volume.

## Graphical abstract

Two cycles of IC is recommended before radiotherapy for patients with LANPC if the initial metastatic cervical lymph node volume is not dominating. Otherwise, three cycles of IC is recommended to further reduce the cervical node volume.

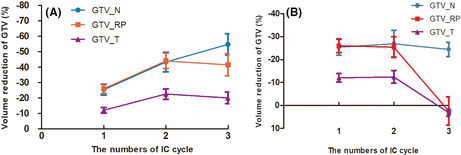

## 1 INTRODUCTION

Nasopharyngeal carcinoma (NPC), known to be endemic in Southern China and Southeast Asia, is prone to recurrence and metastasis.^1^ The anatomic location of NPC does not lend surgery as a viable management option. Taking advantage of the high sensitivity of nasopharyngeal carcinoma cells to radiation, radiotherapy (RT) has become an effective and dominant definitive therapy for NPC, with over 90% of early stage NPC experiencing overall survival (OS) at 5 years. However, for LANPC, the 5‐year OS was only between 41% and 63%.^2^, ^3^ In Mainland China, about 75% of newly diagnosed NPC are LANPC.^4^ Therefore, there remains a compelling need to improve the outcomes of LANPC.

With the development of radiotherapy technology, the local control of LANPC has been greatly enhanced, with distant metastasis has become the major mode of treatment failure for NPC.^5^ According to meta‐analyses of randomized trials, the combination of RT and chemotherapy reduces mortality by 18% and increases the 5‐year OS by 4%–6%.^6^ Among regimens, concurrent chemoradiotherapy (CCRT) has shown OS benefits and has become the standard of care for LANPC.^7^, ^8^

Regarding induction chemotherapy (IC), a meta‐analysis revealed that the addition of IC to CCRT reduces distant failure in LANPC compared with CCRT alone.^9^ Additionally, some studies also showed that the benefit of IC did not translate into an increase in OS.^10^, ^11^, ^12^ However, a publication with the largest sample size and the longest follow‐up period suggested that IC is a better treatment model for NPC patients with clinically staged T_1‐2_/N_2‐3_ and T_3‐4_/N_1‐3_ diseases, especially in combination with CCRT.^13^

Because of the above clinical evidence, IC has been widely used in the treatment of patients with LANPC. However, the optimal number of cycles of IC has not been studied and hence remains controversial. In previous studies from different institutions,^10^, ^12^, ^14^, ^15^, ^16^, ^17^, ^18^ LANPC patients received 2 or 3, or even 4 cycles of IC. Despite the benefits, IC also has its own potential drawbacks, such as causing a delay in radiotherapy, leading to accelerated reproliferation of tumor cells, and yielding reduced radiotherapy tolerance due to adverse reactions of chemotherapy.^19^ The delayed start of radiotherapy may reduce the survival rate.^20^, ^21^, ^22^ Therefore, optimal numbers of IC cycles for different stages of NPC and the best interventional time for radiotherapy are important problems to investigate for prolonging the survival of patients with LANPC.

In this work, we analyzed the optimal number of IC cycles from the time pending for radical radiotherapy. All patients with LANPC underwent multiple computed tomography (CT) scans, obtained the imaging data before and during the IC period, we analyzed the pattern of tumor reduction, dynamically monitored the effects of IC, and objectively evaluated the optimal number of IC cycles. This study provides a basis for the optimization of radiotherapy and chemotherapy combined therapy for LANPC.

## 2 MATERIALS AND METHODS

### 2.1 Patient characteristics

From December 2017 to September 2018, 54 patients with pathologically confirmed nonkeratinizing NPC‐undifferentiated type were retrospectively analyzed for this study.

The inclusion criteria were the completion of a pretreatment evaluation including physical examination, chest CT, contrast‐enhanced nasopharyngeal/neck magnetic resonance imaging (MRI), abdominal ultrasonography, and a bone scan; T_3‐4_ and/or N_2‐3_, M_0_ stage; III/IV stage based on 7th edition UICC (International Union against Cancer/American Joint Committee on Cancer); age between 18 and 70 years old; and Eastern Cooperative Oncology Group (ECOG) score of 0 to 1.

### 2.2 Induction chemotherapy

All patients received CCRT, prior to which they all were given a total of three cycles of platinum‐based IC, once every 3 weeks. Available IC regimens included DP (docetaxel 75 mg/m^2^/d on day 1, cisplatin 25 mg/m^2^/d on days 1–3) or PF (5‐fluorouracil 800–1000 mg/m^2^, continuous intravenous infusion for 96 h, cisplatin 25 mg/m^2^/d on days 1–3). Three cycles of induction chemotherapy with PF (dose is the same as above) were administered in 18 patients, and the other 36 patients underwent DP. Concurrent chemotherapy consisted of cisplatin (30–45 mg/m^2^, intravenous infusion on day 1) given weekly during radiotherapy.

### 2.3 Imaging acquisition and registration

Immobilization was achieved using individualized custom thermoplastic masks encompassing the head, neck, and shoulders in the supine position. Before IC, a CT scan was acquired on a Brilliance Big Bore CT simulator (Philips Inc., Cleveland, OH, USA) with intravenous contrast. Each patient then underwent CT scans on the same CT simulator 14 days after each cycle of chemotherapy following the same protocol except without intravenous contrast. All scans were acquired with a 0.5 × 0.5 mm axial pixel size and a 2.5 mm slice thickness from the vertex to 2 cm caudal to the sternal manubrium. In this article, the three post‐IC CT image sets are denoted as CT_n_ (*n* = 1, 2, 3) for the three cycles of IC, respectively, and the pre‐IC CT image set is denoted as CT_0_. All scanned images were transferred to the MIM system (MIM Software Inc.) for image registration and target delineation.

### 2.4 Definition of target volume

The four CT image sets of the same patient together with their respective diagnostic CT and MRI images were automatically registered in the MIM software and then adjusted manually when necessary. In order to reduce the interobserver variability, the GTVs in each CT image set were contoured by a single trained head and neck radiation oncologist using the MIM software system. In case of doubt, the agreement of another senior radiation oncologist was obtained.

Target volumes were delineated manually according to the NPC intensity‐modulated radiotherapy (IMRT) protocol of our institution. The GTV of the primary tumor (GTV_T_n_), involved cervical lymph nodes (GTV_N_n_), and retropharyngeal lymph node (GTV_RP_n_, *n* = 0, 1, 2, 3) was delineated.

### 2.5 Calculations of tumor response

The study recorded and calculated volume changes during IC. The GTV volumes of the primary tumor and involved lymphatics were evaluated using the contours on the corresponding CT image sets and recorded. The percent change after varying cycles of IC was calculated as follows: (V_CTn_−V_CT0_)/V_CT0_ × 100% (*n* = 1,2,3), where V_CTn_ represents the GTV volume on the CT image, and V_CT0_ is the target volume in the primary CT taken before the IC.

The coordinates of the center of mass (COM) in three orthogonal directions of the GTV, left–right (LR), cranial‐caudal (CC), and anterior–posterior (AP) directions were automatically calculated. In each direction, three displacements could be obtained for each GTV, from the pre‐IC image to each of the post‐IC image.^23^ The three‐dimensional (3D) vector of displacement, which combines errors recorded along all three axes, was defined as the square root of the DLR^2^, DCC^2^, and DAP^2^ mean errors (DLR, DCC, and DAP were the displacements in the LR, CC, and AP directions, respectively).

### 2.6 Statistical analysis

The changes of each GTV volume during IC were analyzed using the Wilcoxon signed‐rank test, with *p* < 0.05 meaning statistically significant changes. The GTV volume reduction was also analyzed against bodyweight change during the IC period using linear regression analysis. The Statistical Package for the Social Sciences (SPSS version 26; IBM Corporation) and Microsoft Office Excel (Microsoft Corporation, Redmond) were used.

## 3 RESULTS

### 3.1 Clinical characteristics

Fifty‐four LANPC patients were analyzed in this study, all managed from December 2017 to September 2018, with a median age of 47 years (range = 21–66). The detailed patient characteristics are shown in Table 1.

**TABLE 1:** Clinical characteristics of the study patients.

Characteristics	*N* (%) (*N* = 54)
Gender	
Male	36 (66.7)
Female	18 (33.3)
Age	
Median (range)	47 (21–66)
NPC histology	
Nonkeratinizing undifferentiated	54 (100.0)
EBER status	
Positive	54 (100.0)
Negative	0
T stage	
T2	9 (16.7)
T3	33 (61.1)
T4	12 (22.2)
*N* stage	
N2	45 (83.3)
N3	9 (16.7)
ECOG performance status	
0	42 (77.8)
1	12 (21.0)

Abbreviations: *N*, number; NPC, nasopharyngeal carcinoma.

### 3.2 Volume reduction following IC

The GTV volume reduction following IC was found to have distinct magnitudes for GTV_T, GTV_RP, and GTV_N, and across different patients, as detailed below. No significant differences were apparent in the mean volume reduction of the gross tumor or nodes between those who received 3 cycles of PF (*n* = 18) versus DP (*n* = 36).

#### 3.2.1 During IC, the gross tumor volume of the primary lesion gradually shrinks (Figures 1 and 2) but tapers off after 2 cycles

**FIGURE 1:**
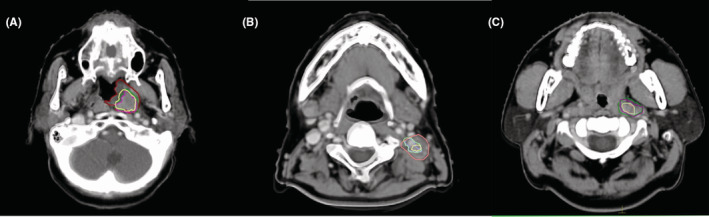
The primary tumor's gross tumor volume as well as that of the cervical and retropharyngeal lymph nodes on pretreatment CT and three repeat CTs. (A) Primary tumor: the red, yellow, green, rosy lines; (B) cervical lymph nodes: the orange, green, purple, yellow lines; (C) and retropharyngeal lymph nodes: the green, purple, pink, light green lines denote the gross tumor volumes on pretreatment CT and the first, second, and third repeat CTs, respectively. CT, computed tomography.

**FIGURE 2:**
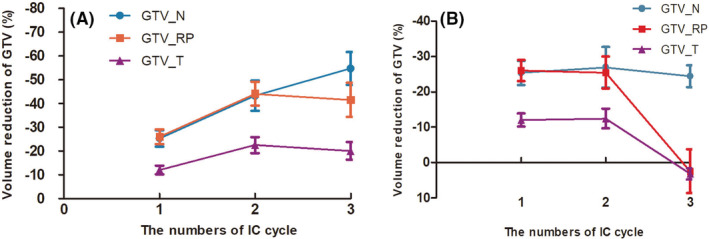
The volume reductions in GTV_T, GTV_N, and GTV_RP. (A) The volume reduction of GTVs in IC was calculated as follows: (VCTn‐VCT0)/VCT0 × 100% (*n* = 1, 2, 3); (B) GTVs volume reduction following different numbers of IC cycle, was calculated as follows: (VCTn‐VCTn‐1)/VCTn‐1 × 100% (*n* = 1, 2, 3); using the initial volume on pretreatment CT as the reference, VCTn was the volume on repeated CT and VCT0 for the pretreatment CT. The mean values are displayed by the given lines, with the error bars corresponding to the 95% confidence intervals. GTV_T, gross tumor volume of the primary tumor; CTV_N, cervical lymph nodes; GTV_RP, retropharyngeal lymph nodes; IC, induction chemotherapy.

Compared with the initial volume on pre‐IC CT (GTV_T_0_), the total volume reduction of GTV_T_n_ (*n* = 1, 2, 3) after each IC cycle was −3.1 ± 0.7 cc (12.0%), 7.0 ± 1.9 cc (22.5%), and 6.9 ± 2.0 cc (20.1%), respectively. GTV_T significantly decreased its volume following the first two IC cycles (*p* < 0.05), but not after the third cycle (Table 2).

**TABLE 2:** The volume reduction of GTVs.

Target	Cycle of IC	Volume reduction (cc)	Percent volume reduction (%)	*p*
GTV_T	1	−3.1 ± 0.7	12.0	<0.05^a^
	2	−7.0 ± 1.9	22.5	<0.05^b^
	3	−6.9 ± 2.0	20.1	>0.05^c^
GTV_N	1	−7.3 ± 1.7	25.3	<0.05^a^
	2	−13.5 ± 2.9	43.2	<0.05^b^
	3	−17.2 ± 3.4	54.7	<0.05^c^
GTV_RP	1	−1.4 ± 0.4	26	<0.05^a^
	2	−2.5 ± 0.5	44.1	<0.05^b^
	3	−2.4 ± 0.5	42.2	>0.05^c^

Abbreviations: AP, anterior–posterior; GTV_N, gross tumor volume of cervical lymph nodes; GTV_RP, gross tumor volume of retropharyngeal lymph nodes; GTV_T, gross tumor volume of the primary tumor; IC, induction chemotherapy; LR, left–right; CC, cranial–caudal.

^a^ The volume after the first cycle of compared with before IC.

^b^ The volume after the second cycle of compared with before IC.

^c^ The volume after the third cycle of compared with before IC.

#### 3.2.2 During IC, the size of cervical lymph node metastases continues to shrink following each cycle, at reduction rates higher than those of primary nasopharyngeal lesions (Figures 1 and 2)

Using the initial volume on pre‐IC CT (GTV_N_0_) as a comparison, the total volume reduction of GTV_N_n_ (*n* = 1, 2, 3) after each IC cycle was 7.3 ± 1.7 cc (25.3%), 13.5 ± 2.9 cc (43.2%), and 17.2 ± 3.4 cc (54.7%), respectively (Table 2). For GTV_N, the volume reduction was significant (*p* < 0.05) following all three IC cycles, with a single‐cycle GTV_N relative volume reduction rate of 25.3%, 26.9%, and 24.4% respectively after each cycle of IC.

#### 3.2.3 The volume change trend of retropharyngeal lymph nodes is similar to primary nasopharyngeal lesions (Figures 1 and 2)

Compared with the initial volume on pre‐IC CT (GTV_RP_0_), the volume changes of GTV_RP_n_ (*n* = 1, 2, 3) after IC was 1.4 ± 0.4 cc (26.0%), 2.5 ± 0.5 cc (44.1%), and 2.4 ± 0.5 cc (42.2%), respectively. Significant volume reduction occurred only in the first two cycles (*p* < 0.05), and after the third cycle, the volume even slightly increased (Table 2).

### 3.3 Displacement of COM

The displacements of all GTVs are shown in Table 3. The average COM displacements of each of the GTV_T, GTV_N, and GTV_RP were all less than 1.5 mm in individual orthogonal directions, and their mean 3D displacements were 2.6, 4.0, and 1.7 mm, respectively (Table 3). We also noticed that the largest magnitude of displacements of lymph node GTVs was in the CC direction, with most of them shifted toward the cranial direction.

**TABLE 3:** Description of mass displacements of the target volumes (mm).

Target	Direction	Mean	System error	Random error	3D vector displacement of target volumes
GTV_T	LR	−1.5	2.7	1.19	2.6 ± 2.5
	CC	−0.2	0.9	0.43	
	AP	−1	1	0.7	
GTV_N	LR	0.6	2.6	2.44	4.0 ± 3.1
	CC	0.8	3.5	2.4	
	AP	0.5	2.6	2.3	
GTV_RP	LR	−0.74	0.69	0.64	1.7 ± 0.5
	CC	−1.4	2.2	1.3	
	AP	−0.6	0.9	0.63	

*Note*: Data are illustrated with the format of mean ± standard deviation, with the 95% confidence interval in parentheses. A negative number denotes a shift to the right, caudal, and posterior in the LL, CC, and AP directions, respectively.

Abbreviations: 3D, three‐dimensional; AP, anterior–posterior; CC, cranial–caudal; GTV_N, gross tumor volume of cervical lymph nodes; GTV_RP, gross tumor volume of retropharyngeal lymph nodes; GTV_T, gross tumor volume of the primary tumor; LR, left–right.

### 3.4 Volume reduction and bodyweight change

During IC, the body weight change of the patients ranged from −10.2% to 11.6%. The average body weight increased 3.1%, 3.1%, and 1.6% respectively after each cycle of chemotherapy. The volume reductions of the GTVs do not have a strong correlation with weight change, which makes it hard to predict GTV volume reduction from the bodyweight change.

### 3.5 Adverse reactions during IC

The most common side effects during IC were myelosuppression, mainly leukopenia and neutropenia, and no neutropenia fever. Most of the patients experienced grade I‐II myelosuppression. Only one patient developed grade III myelosuppression after three cycles of IC which improved after being treated with recombinant human granulocyte colony‐stimulating factor. Another common reaction was gastrointestinal reaction, mainly nausea and vomiting, including 48 cases of grade I gastrointestinal reaction and 6 cases of grade II delayed vomiting. After strengthening symptomatic support treatment, all patients completed three cycles of IC. Table 4 describes the toxicity details.

**TABLE 4:** Acute events of induction chemotherapy.

	No. (%)			
	Any Grade	Grade 1	Grade 2	Grade ≥ 3
Neutropenia	33 (61.0)	27 (50.0)	3 (5.5)	3 (5.5)
leucopenia	33 (61.0)	27 (50.0)	3 (5.5)	3 (5.5)
Anemia	12 (22.0)	12 (22.0)	0 (0.0)	0 (0.0)
Thrombocytopenia	3 (6.0)	3 (6.0)	0 (0.0)	0 (0.0)
Nausea/vomiting	54 (100.0)	48 (89.0)	6 (11.0)	0 (0.0)
Asthenia	54 (100.0)	54 (100.0)	0 (0.0)	0 (0.0)
Diarrhea/constipation	54 (100.0)	54 (100.0)	0 (0.0)	0 (0.0)
Neurotoxicity	0 (0.0)	0 (0.0)	0 (0.0)	0 (0.0)
Hepatotoxicity	9 (17.0)	9 (17.0)	0 (0.0)	0 (0.0)

### 3.6 Impact on subsequent CCRT

All patients in this study completed 3 cycles of induction chemotherapy prior to concurrent chemoradiotherapy. All patients received 5–7 cycles of weekly cisplatin for current chemotherapy during radiation treatment. The completion rate was 100%.

## 4 DISCUSSION

The patients enrolled in this study were clinically classified as T_3‐4_ or N_2_. According to Du^24^ and Lan,^25^ these patients are all in the high‐risk group for local recurrence and distant metastasis and can benefit from IC.

With the imaging data in this study, the optimal number of IC cycles was investigated and identified for the first time. Our study showed that the GTVs of LANPC changed relatively substantially throughout IC. The effective rate was 89% with only six cases failing to show a tumor reduction after IC. The results of our study suggest that GTV_T continues to shrink after the first two cycles of IC. However, further tumor reduction was not significant after the third cycle and some patients even showed slight volume increases, especially those patients with T4 stage. Our data analysis showed that 27 (50%) of the 54 cases experienced a GTV_T increase after the third cycle of IC. The volume change of retropharyngeal lymph nodes shows the same trend as GTV_T. After the third cycle of IC, the volume of retropharyngeal lymph nodes remained unchanged or even slightly increased.

Volume reductions of the GTV during IC have been previously noted. Li et al. showed a relative volume reduction of 12.7% in GTV_T after IC in nonmetastatic NPC patients.^26^ Zheng et al. reported a mean volume regression of 23.1% in GTV_T and 33.1% in GTV_N among 44 NPC patients who received two cycles of IC.^27^ Lee et al. reported that in 20 cases of advanced nasopharyngeal carcinoma, 35% of patients had achieved significant downstaging.^28^ In their study, significant GTV_T volume reductions were observed, with the mean reduction being 61.4% (range = 36.7–83.6%), with 70% of patients having experienced a >50% reduction in GTV_T following IC. In addition, He et al. compared survival outcomes from patients treated with two, three, and four IC cycles and found similar survival between two and three cycles IC groups, while four cycles IC was associated with worse overall survival and higher incidence of treatment‐related toxicities.^29^ The findings of our study showed tumor reduction in magnitudes to a certain extent consistent with the results of the above clinical studies. However, in clinical practice, further shrinkage in the gross tumor and lymph nodes may not meaningfully correspond with outcomes; rather, it is likely that the degree of benefit is limited to a subset of patients. Therefore, judging from our results, we recommend that for patients with stage T_3‐4_ or large retropharyngeal lymph nodes, two cycles of IC are optimal, and radiotherapy should be initiated after two IC cycles. The delay of radiation therapy may undermine the efficacy of IC, and further increases in the number of IC cycles may not benefit the patients.

Our study also shows that the volume of GTV_N steadily decreased after all three IC cycles and exhibited a greater reduction in contrast to GTV_T and GTV_RP. The single‐cycle volume reduction of GTV_N after each IC cycle was statistically significant. After three cycles of induction, the volume of GTV_N decreased by an average of 55%. This is consistent with the results of several other publications. With NPC patients included, these head and neck patients showed average GTV_N volume reduction in the range of 33%–68%.^27^, ^30^ A study also reported that the number of IC appeared to be an independent predictor and associated with an improvement in survival. For N2‐3 NPC, survival data of the 4 cycles of IC were better than those of 2 cycles.^31^ In our study, all but one patient showed a GTV_N volume reduction after three cycles of IC. The exception patient even showed a GTV_N volume increase. The clinical stage of this patient was T_3_N_2_M_0_ stage III. For this patient, the right cervical lymph node continued to enlarge after IC, and the GTV_T also increased after the second cycle of IC. The most likely explanation was the primary drug resistance in this patient.

In a sense, the COM displacement was used to gauge the tumor response, since the degree of response impacts the location of the remaining disease. There are little data published about the position variations of LANPC of IC. In our study, the average COM displacement of all GTVs was less than 1.5 mm in all three orthogonal directions, and the average 3D vector displacements ranged from 1.7–4.0 mm. The GTV_T displacements were larger in the LR and AP directions relative to the CC direction. This could be because the primary tumor receded from the adjacent air cavity as well as a shift in the posterior portion of the disease abutting the relatively stable vertebrae and paravertebral muscles (as shown in Figure 1A). However, the displacements of the cervical lymph nodes were more considerable in the CC direction. We also found that the cervical lymph nodes (except for one patient whose GTV_T increased during IC) moved in the lateral–medial direction. The left and right sides of GTV_N shifted medially during IC by an average of 0.8 mm and 0.4 mm, respectively. While the GTV displacement results obtained in this study is not directly useful during IC, they may be important for CCRT if similar trends also follow chemotherapy concurrent to radiation therapy.

To our knowledge, this is the first time that GTV volume reduction was explored through imaging in effort to optimize IC delivery for LANPC. However, there are three major limitations. First, repeat CTs were done without contrast, which may not be of sufficient quality in order to allow for accurate target definition. Although it is logical to use contrast on each scan, frequent contrast enhancement may lead to a risk of renal impairment and will also increase the financial burden for patients. Second, because surgery is not standard for NPC, it is hard to obtain the pathological remission rate, this study focused on the trend of tumor volume change to optimize the number of IC cycles, and only investigated the completion rate of the subsequent CCRT. Long‐term follow‐up may be studied in future studies. Finally, even though our cohort had only 54 patients, our findings were able to show the changes in tumor shrinkage, and then provide evidence and new ideas for clinical practice. Because the sample size was only 54 patients, our conclusion remains to be validated in large‐scale studies. Further large‐sample with long‐term follow‐up and a prospective clinical trial is warranted.

## 5 CONCLUSION

Three cycles of platinum‐based doublet IC were well tolerated and effective for LANPC patients. The nasopharyngeal carcinoma patients with the same stage (III and IV) but different T and N stages may benefit from different cycles of IC. Volume reductions in the primary tumor and retropharyngeal lymph nodes are similar, based on which two cycles of IC are recommended for patients with large volumes of these targets. However, cervical lymph nodes showed continuous and significant volume reductions following all three cycles of IC. Therefore, a three‐cycle IC course are recommended for patients with large cervical lymph node volumes.

## AUTHOR CONTRIBUTIONS

**Ying Li:** Data curation (lead); funding acquisition (equal); methodology (equal); writing – original draft (lead). **Jianping Bi:** Data curation (equal); methodology (equal); writing – original draft (equal). **Guoliang Pi:** Investigation (equal). **Hanping He:** Data curation (equal); formal analysis (equal). **Yanping Li:** Data curation (equal). **Dandan Zheng:** Writing – review and editing (equal). **Zecheng Wei:** Investigation (equal). **Guang Han:** Conceptualization (lead); data curation (equal); funding acquisition (equal); methodology (lead); writing – review and editing (lead).

## FUNDING INFORMATION

This work was supported by Hubei Provincial Health Commission (grant no. WJ2017M145 and ZY2021M008).

## CONFLICT OF INTEREST STATEMENT

The authors declare that they have no conflict of interest to disclose.

## ETHICS APPROVAL AND CONSENT TO PARTICIPATE

The Medical Ethics Committee of Hubei Cancer Hospital, Tongji Medical College, Huazhong University of Science and Technology approved this study. Informed consent was obtained from all patients.

## PATIENT CONSENT FOR PUBLICATION

Not applicable.

## Data Availability

The data sets generated from this study are all included in the manuscript and figures.

## References

[1] Bray F , Ferlay J , Soerjomataram I , Siegel RL , Torre LA , Jemal A . Global cancer statistics 2018: GLOBOCAN estimates of incidence and mortality worldwide for 36 cancers in 185 countries. CA Cancer J Clin. 2018;6:394‐424. doi:10.3322/caac.21492 30207593

[2] Xiao WW , Han F , Lu TX , Chen CY , Huang Y , Zhao C . Treatment outcomes after radiotherapy alone for patients with early‐stage nasopharyngeal carcinoma. Int J Radiat Oncol Biol Phys. 2009;74:1070‐1076. doi:10.1016/j.ijrobp.2008.09.008 19231110

[3] Cao XP , Lu TX , Ye WJ , Cui NJ . Prospective study on long‐term efficacy of external plus intracavitary radiotherapy on stage I–II nasopharyngeal carcinoma. Ai Zheng. 2007;26:204‐207. 17298754

[4] Ho FC , Tham IW , Earnest A , Lee KM , Lu JJ . Patterns of regional lymphnode metastasis of nasopharyngeal carcinoma: a meta‐analysis of clinical evidence. BMC Cancer. 2012;12:98. doi:10.1186/1471-2407-12-98 22433671 PMC3353248

[5] Sun X , Su S , Chen C , et al. Long‐term outcomes of intensity‐modulated radiotherapy for 868 patients with nasopharyngeal carcinoma: an analysis of survival and treatment toxicities. Radiother Oncol. 2014;110:398‐403. doi:10.1016/j.radonc.2013.10.020 24231245

[6] Al‐Sarraf M , Reddy MS . Nasopharyngeal carcinoma. Curr Treat Options Oncol. 2002;3:21‐32. doi:10.1007/s11864-002-0038-8 12057084

[7] Al‐Sarraf M , LeBlanc M , Giri PG , et al. Chemoradiotherapy versus radiotherapy in patients with advanced nasopharyngeal cancer: phase III randomized intergroup study 0099. J Clin Oncol. 1998;16:1310‐1317. doi:10.1200/JCO.1998.16.4.1310 9552031

[8] Lee AW , Tung SY , Chua DT , et al. Randomized trial of radiotherapy plus concurrent‐adjuvant chemotherapy vs radiotherapy alone for regionally advanced nasopharyngeal carcinoma. J Natl Cancer Inst. 2010;102:1188‐1198. doi:10.1093/jnci/djq258 20634482

[9] OuYang PY , Xie C , Mao YP , et al. Significant efficacies of neoadjuvant and adjuvant chemotherapy for nasopharyngeal carcinoma by meta‐analysis of published literature‐based randomized, controlled trials. Ann Oncol. 2013;24:2136‐2146. doi:10.1093/annonc/mdt146 23613477

[10] Tan T , Lim WT , Fong KW , et al. Concurrent chemo‐radiation with or without induction gemcitabine, carboplatin, and paclitaxel: a randomized, phase 2/3 trial in locally advanced nasopharyngeal carcinoma. Int J Radiat Oncol Biol Phys. 2015;91:952‐960. doi:10.1016/j.ijrobp.2015.01.002 25832687

[11] Xu TT , Zhu GP , He XY , et al. A phase III randomized study comparing neoadjuvant chemotherapy with concurrent chemotherapy combined with radiotherapy for locoregionally advanced nasopharyngeal carcinoma: updated long‐term survival outcomes. Oral Oncol. 2014;50:71‐76. doi:10.1016/j.oraloncology.2013.11.002 24315404

[12] Lim AM , Corry J , Collins M , et al. A phase II study of induction carboplatin and gemcitabine followed by chemoradiotherapy for the treatment of locally advanced nasopharyngeal carcinoma. Oral Oncol. 2013;49:468‐474. doi:10.1016/j.oraloncology.2012.12.012 23369852

[13] Hao P , Chen L , Jun M , et al. Induction chemotherapy improved long‐term outcomes of patients with Locoregionall advanced nasopharyngeal carcinoma: a propensity matched analysis of 5‐year survival outcomes in the era of intensity‐modulated radiotherapy. J Cancer. 2017;8(3):371‐377. doi:10.7150/jca.16732 28261337 PMC5332887

[14] Blanchard P , Lee A , Marguet S , et al. Chemotherapy and radiotherapy in nasopharyngeal carcinoma: an update of the MAC‐NPC meta‐analysis. Lancet Oncol. 2015;16:645‐655. doi:10.1016/S1470-2045(15)70126-9 25957714

[15] Hui EP , Ma BB , Leung SF , et al. Randomized phase II trial of concurrent cisplatin radiotherapy with or without neoadjuvant docetaxel and cisplatin in advanced nasopharyngeal carcinoma. J Clin Oncol. 2009;27:242‐249. doi:10.1200/JCO.2008.18.1545 19064973

[16] Chi KH , Chang YC , Guo WY , et al. A phase III study of adjuvant chemotherapy in advanced nasopharyngeal carcinoma patients. Int J Radiat Oncol Biol Phys. 2002;52:1238‐1244. doi:10.1016/s0360-3016(01)02781-x 11955734

[17] Kong L , Zhang YW , Hu CS , Guo Y , Lu JJ . Effects of induction docetaxel, platinum, and fluorouracil chemotherapy in patients with stage III or IVA/B nasopharyngeal cancer treated with concurrent chemoradiation therapy: final results of 2 parallel phase clinical trial. Cancer. 2017;123:2258‐2267. doi:10.1002/cncr.30566 28192641

[18] Zhang Y , Chen M , Chen C , Kong L , Lu JJ , Xu B . The efficacy and toxicities of intensive induction chemotherapy followed by concurrent chemotherapy in nasopharyngeal carcinoma patients with N3 disease. Sci Rep. 2017;7:3668. doi:10.1038/s41598-017-03963-8 28623353 PMC5473919

[19] Sun Y , Li WF , Chen NY , et al. Induction chemotherapy plus concurrent chemotherapy versus concurrent chemoradiotherapy alone in locoregionally advanced nasopharyngeal carcinoma: a phase 3, multicentre, randomized controlled trial. Lancet Oncol. 2016;12:1509‐1520. doi:10.1016/S1470-2045(16)30410-7 27686945

[20] Davis AJ , Tannock JF . Repopulation of tumour cells between cycles of chemotherapy: a neglected factor. Lancet Oncol. 2000;1:86‐93. doi:10.1016/s1470-2045(00)00019-x 11905673

[21] Chen YP , Mao YP , Zhang WN , et al. Prognostic value of wait time in nasopharyngeal carcinoma treated with intensity modulated radiotherapy: a propensity matched analysis. Oncotarget. 2016;7:14973‐14982. doi:10.18632/oncotarget.7789 26942870 PMC4924766

[22] Liang H , Xiang YQ , Lu X , et al. Survival impact of waiting time for radical radiotherapy in nasopharyngeal carcinoma: a large institution‐based cohort study from an endemic area. Eur J Cancer. 2017;73:48‐60. doi:10.1016/j.ejca.2016.12.009 28161498

[23] Van Herk M . Errors and margins in radiotherapy. Semin Radiat Oncol. 2004;14:52‐64. doi:10.1053/j.semradonc.2003.10.003 14752733

[24] Du XJ , Tang LL , Chen L , et al. Neoadjuvant chemotherapy in locally advanced nasopharyngeal carcinoma: defining high‐risk patients who may benefit before concurrent chemotherapy combined with intensity‐modulated radiotherapy. Sci Rep. 2015;5:16664. doi:10.1038/srep16664 26564805 PMC4643258

[25] Lan XW , Xiao Y , Zou XB , Zhang XM , OuYang PY , Xie FY . Outcomes of adding induction chemotherapy to concurrent chemoradiotherapy for stage T3N0‐1 nasopharyngeal carcinoma: a propensity‐matched study. Onco Targets Ther. 2017;10:3853‐3860. doi:10.2147/OTT.S133917 28814884 PMC5546817

[26] Li S , Shen L . The change in tumor volume after induction chemotherapy with docetaxel plus cisplatin in 259 nasopharyngeal carcinoma patients. Eur Arch Otorhinolaryngol. 2021;8:3027‐3035. doi:10.1007/s00405-020-06477-8 33386968

[27] Zheng L , Liao W , Xu P , Li B , Wen H , Zhang S . Tumor volume reduction after gemcitabine plus cisplatin induction chemotherapy in locally advanced nasopharyngeal cancer: comparison with paclitaxel and cisplatin regimens. Med Sci Monit. 2018;24:8001‐8008. doi:10.12659/MSM.909736 30406770 PMC6237045

[28] Lee AW , Lau KY , Hung WM , et al. Potential improvement of tumor control probability by induction chemotherapy for advanced nasopharyngeal carcinoma. Radiother Oncol. 2008;87(2):204‐210. doi:10.1016/j.radonc.2008.02.003 18329742

[29] He Y , Zhao Z , Wang Y , et al. Optimizing number of cycles of induction chemotherapy for patients with nasopharyngeal carcinoma: retrospective survival analysis. Head Neck. 2020;42:2067‐2076. doi:10.1002/hed.26141 32202686

[30] Wang L , Wu Z , Xie D , et al. Reduction of target volume and the corresponding dose for the tumor regression field after induction chemotherapy in Locoregionally advanced nasopharyngeal carcinoma. Cancer Res Treat. 2019;51:685‐695. doi:10.4143/crt.2018.250 30121968 PMC6473261

[31] Wei J , Feng H , Xiao W , et al. Cycle number of neoadjuvant chemotherapy might influence survival of patients with T1‐4N2‐3M0 nasopharyngeal carcinoma. Chin J Cancer Res. 2018;1:51‐60. doi:10.21147/j.issn.1000-9604.2018.01.06 PMC5842233 29545719

